# Investigation of Reference Genes in *Vibrio parahaemolyticus* for Gene Expression Analysis Using Quantitative RT-PCR

**DOI:** 10.1371/journal.pone.0144362

**Published:** 2015-12-11

**Authors:** Yue-jiao Ma, Xiao-hong Sun, Xiao-yan Xu, Yong Zhao, Ying-jie Pan, Cheng-An Hwang, Vivian C. H. Wu

**Affiliations:** 1 College of Food Science and Technology, Shanghai Ocean University, Shanghai, People's Republic of China; 2 Shanghai Engineering Research Center of Aquatic Product Processing & Preservation, Shanghai 201306, China; 3 Key Laboratory of Exploration and Utilization of Aquatic Genetic Resources, Shanghai Ocean University, Ministry of Education, Shanghai, People's Republic of China; 4 Residue Chemistry and Predictive Microbiology Research Unit, Eastern Regional Research Center, Agricultural Research Service, United States Department of Agriculture, Wyndmoor, PA, 19038, United States of America; 5 The Pathogenic Microbiology Laboratory, School of Food and Agriculture, University of Maine, Orono, ME, 04469–5735, United States of America; 6 Produce Safety and Microbiology Research Unit, Western Regional Research Center, Agricultural Research Service, United States Department of Agriculture, Albany, CA, 94710, United States of America; Northwestern University, UNITED STATES

## Abstract

*Vibrio parahaemolyticus* is a significant human pathogen capable of causing foodborne gastroenteritis associated with the consumption of contaminated raw or undercooked seafood. Quantitative RT-PCR (qRT-PCR) is a useful tool for studying gene expression in *V*. *parahaemolyticus* to characterize its virulence factors and understand the effect of environmental conditions on its pathogenicity. However, there is not a stable gene in *V*. *parahaemolyticus* that has been identified for use as a reference gene for qRT-PCR. This study evaluated the stability of 6 reference genes (*16S rRNA*, *recA*, *rpoS*, *pvsA*, *pvuA*, and *gapdh*) in 5 *V*. *parahaemolyticus* strains (O3:K6-clinical strain-*tdh* ^+^, ATCC33846-*tdh* ^+^, ATCC33847-*tdh* ^+^, ATCC17802-*trh* ^+^, and F13-environmental strain*-tdh* ^+^) cultured at 4 different temperatures (15, 25, 37 and 42°C). Stability values were calculated using GeNorm, NormFinder, BestKeeper, and Delta CT algorithms. The results indicated that *recA* was the most stably expressed gene in the *V*. *parahaemolyticus* strains cultured at different temperatures. This study examined multiple *V*. *parahaemolyticus* strains and growth temperatures, hence the finding provided stronger evidence that *recA* can be used as a reference gene for gene expression studies in *V*. *parahaemolyticus*.

## Introduction

*Vibrio parahaemolyticus* is a Gram-negative halophilic pathogenic bacterium [1]. It thrives in warm climates in marine or estuarine environment and is frequently associated with raw or undercooked seafood [2]. The bacterium has been recognized as a major cause of foodborne gastroenteritis linked to seafood consumption throughout the world [1–3].

*V*. *parahaemolyticus* is ubiquitous, but its survival in the environment mainly depends on the environmental temperature [1, 4]. Studies have indicated that temperature regulates the expression of virulence gene in pathogenic bacteria [5–7]. Due to the warming of ocean temperature, *V*. *parahaemolyticus* has been detected in coastal waters as far north as the southern coast of Alaska [8]. Under changing environmental conditions, the gene expression of *V*. *parahaemolyticus* may change and allow the bacterium to survive, maintain normal cellular functions, and adapt its transcriptome to a new steady state.

Quantitative reverse transcription polymerase chain reaction (qRT-PCR) is widely used to quantify and compare the levels of gene transcription for its high sensitivity, accuracy, and reproducibility [9, 10]. Factors such as RNA quality and the efficiencies of reverse transcription and PCR may affect the accuracy and reliability of qRT-PCR, therefore results are typically “normalized” by comparing to one or more internal reference genes to eliminate sample to sample variation. The internal reference gene must be stably expressed since variation in reference gene expression can lead to false results [11]. However, reference genes vary greatly among species and organisms grown under different environmental conditions [12, 13]. Therefore, the identification of stable reference genes is a crucial step in the design of qRT-PCR experiments.

It is unlikely that there is a single universal reference gene suited for all experimental conditions [14–16]. Therefore, it is critical to select reliable reference genes that are consistently expressed under specific experimental conditions for normalization in gene expression analysis. In this study, we evaluated the stability of 6 reference genes (*16S rRNA*, *recA*, *rpoS*, *pvsA*, *pvuA*, and *gapdh*) in 5 strains of *V*. *parahaemolyticus* cultured at four different temperatures. We then identified the most stable internal reference genes for each growth temperature using GeNorm, NormFinder, BestKeeper, and Delta CT algorithms and recommended the most suitable reference genes in *V*. *parahaemolyticus* for transcript analysis using qRT-PCR.

## Materials and Methods

### Selection of candidate genes and primer design

Six candidate reference genes (*16S rRNA*, *recA*, *rpoS*, *pvsA*, *pvuA*, and *gapdh*) were selected from genes previously used in qRT-PCR assays for other bacterial species (Table 1). Primers were designed using Primer5 software (http://frodo.wi.mit.edu/cgi-bin/primer5/primer5_www.cgi) based on the available DNA sequences of *V*. *parahaemolyticus* RIMD 2210633 (GenBank Assembly ID GCA_000196095.1). Primer efficiencies were determined by construction of a standard curve using 10-fold serial dilutions of pooled cDNA template [17]. Primer specificity was determined by melting curve analysis and gel electrophoresis [18].

**Table 1:** Primer information for 6 selected candidate reference genes.

Gene	Forward Primer (5’-3’)	Reverse Primer (5’-3’)	Amplicon size (bp)	Amplification efficiency (%)	Correlation coefficient (R^2^)	Reference
*16S rRNA*	TATCCTTGTTTGCCAGCGAG	CTACGACGCACTTTTTGGGA	186	96.968	0.999	This study
*rpoS*	GACAATGCGTCAGAGACG	GAGGTGAGAAGCCAATTTC	151	104.989	0.991	[28]
*pvsA*	CTCCTTCATCCAACACGAT	GGGCGAGATAATCCTTGT	104	104.206	0.989	[29]
*pvuA*	CAAACTCACTCAGACTCCA	CGAACCGATTCAACACG	156	96.724	0.996	[29]
*gapdh*	TGTTGACGTTGTAGCAGAAG	ACCGAACTTGTCGTTAAGAA	235	99.503	0.995	[24]
*recA*	GCTAGTAGAAAAAGCGGGTG	GCAGGTGCTTCTGGTTGAG	165	100.353	0.988	This study

### *V*. *parahaemolyticus* strains and culture conditions

Five strains of *V*. *parahaemolyticus* were used in this study: O3:K6 (clinical strain), ATCC33846 (*tdh* ^+^/O3), ATCC33847 (*tdh* ^+^/O3), ATCC17802 (*trh* ^+^ /O1), and F13 (*tdh* ^+^, environmental strain). Each strain was preserved in glycerol at -80°C, and, after twice activated in tryptic soy broth(Beijing Land Bridge Technology Co., Beijing, China)with 3% sodium chloride (TSB-3% NaCl) at 37°C, each was grown in TSB-3% NaCl at 15, 25, 37, and 42°C with shaking at160 rpm. These temperatures were chosen for that *V*. *parahaemolyticus* is rarely detected in seawater temperatures at <15°C [1], 37°C is the optimum growth temperature for *V*. *parahaemolyticus* and human body temperature, and 42°C is as a sublethal high-temperature stress [19]. The exponential growth phase in each of the growth curves of these 5 strains was identified by the OD_600 nm_ reading from Bioscreen C MBR (Oy Growth Curves Ab Ltd., Helsinki, Finland). Bacterial cells collected from the exponential growth phase (OD reading ~0.5) were used for total RNA extraction.

### RNA extraction, cDNA synthesis, and qRT-PCR

Total RNA was extracted from cells using a Trizol reagent (Invitrogen, Carlsbad, CA, USA) and quantified using a NanoDrop ND 2000 spectrophotometer (Thermo Fisher Scientific, Waltham, MA, USA). Complementary DNA (cDNA) was synthesized through random hexamer primed reactions using a PrimeScript RT reagent kit with gDNA Eraser (Takara Bio Inc., Otsu, Japan). qRT-PCR reactions were carried out in a 7500 Fast Real-Time PCR system (Applied Biosystems, Waltham, MA, USA). Each reaction contained 10 μL of SYBR *Premix Ex Taq*II (2x) (Takara Bio Inc.), 0.8 μM each forward and reverse primer, and cDNA transcribed from 10 ng RNA. qRT-PCR (25-μl reaction volume) was performed as follows: 95°C for 30 sec followed by 40 cycles at 95°C for 5 sec, 55°C for 34 sec, and 72°C for 45 sec, and then melt curve analysis at 95°C for 15 sec then 60°C for 1 min. Each PCR reaction was conducted in triplicate, and controls without template were included. All measurements were performed in duplicate. In addition, melting curve analysis was performed in each assay in order to detect non-specific amplifications.

### Data analysis

Expression data for the reference genes were obtained in the form of threshold cycle (Ct) values. The amplification efficiencies and correlation coefficients were calculated using ABI 7500 Software v2.0 (Applied Biosystems). The stability of reference genes was evaluated using four software programs: GeNorm v.3.5[15], NormFinder [20], BestKeeper [21], and comparative Delta CT method [22] algorithms. All analyses were carried out using standard setup configurations. Based on the rankings from each program, RefFinder (http://www.leonxie.com/referencegene.php?type=reference) was used to assign an appropriate weight to each gene and calculated the geometric mean of the weights to provide an overall final ranking.

## Results

### Standard curve, PCR efficiency and product specificity

The efficiency of PCR reaction of each candidate reference gene was determined using a 10-fold serial dilution of pooled cDNA. The calculated efficiencies for the candidate reference genes were between 96.724% and 104.989% (Table 1). The efficiency curves for the candidate reference genes were found to be linear with correlation coefficients (R^2^) ranging from 0.988 to 0.999. Melting peak analysis produced a single homogenous peak for all primer sets, indicating specific amplification of a single product with no primer-dimer being observed (Figure A in S1 File). Gel electrophoresis analysis of the amplified products for all primer sets revealed single bands of the expected size (Figure B in S1 File).

### Expression of candidate reference genes in culture

**Fig 1:**
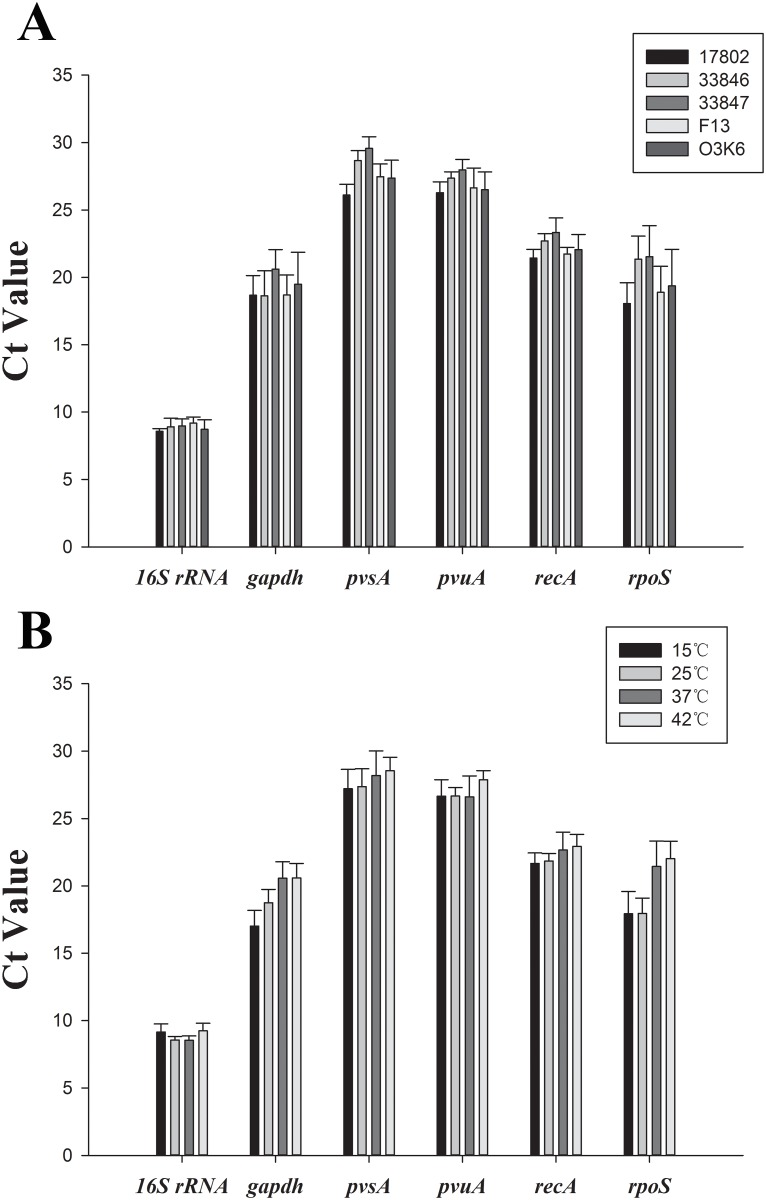
Expression levels of reference genes. (A) Expression of reference genes for 5 *V*. *parahaemolyticus* strains. (B) Expression of reference genes for all culture temperatures. Gene expression levels are represented by average Ct values. Each bar represents the mean Ct values from three independent experiments. Error bars indicate standard deviations.

Using the optimized qRT-PCR conditions, all the genes were analyzed in triplicate for all the strains and temperatures. The expression of candidate genes (mean Ct values) for all temperatures and strains are shown in Fig 1A and 1B, respectively. The 6 candidate genes showed a relatively narrow range of Ct values. The lowest Ct value was 8.15, and the highest was 30.89. The majority of the Ct values were between 8 and 30, with the average Ct value of all candidates across all the strains and growth temperatures at approximately 20.83 (Fig 1A). The *16S rRNA* gene, with a variation (standard deviation, SD) of 1.79, showed the least variation in Ct values among the strains, while *rpoS*, with a variation of 7.66, showed the greatest variation in Ct values. The *16S rRNA* showed the least variation in Ct values across the growth temperatures with a variation of 1.78, while *rpoS* showed the greatest variation in Ct values with a variation of 7.66 (Fig 1B). Across all the strains and growth temperatures, *16S rRNA* gene showed the highest expression with an average Ct of 8.87, while *pvsA* showed the least expression with an average Ct of 27.83. Overall, the expression of candidate genes showed greater variation across the growth temperatures than across the strains.

### Analysis of reference gene expression using BestKeeper, NormFinder, Delta CT, and GeNorm

The BestKeeper, a program based on Microsoft Excel, was used to determine the reference genes with the greatest expression stability. The descriptive statistics of the 6 candidate genes in the 5 *V*. *parahaemolyticus* strains across the different growth temperatures are shown in Table 2. Based on the BestKeeper analysis, the *16S rRNA* gene was ranked as the most stably expressed gene in the 5 *V*. *parahaemolyticus* strains across the different growth temperatures, with a standard deviation (SD) of 0.45. The *recA* gene, with a SD of 0.87, was ranked as the second most stably expressed gene. The *gapdh* and *rpoS* were found to be the least stably expressed genes, with SD of 1.49 and 2.00, respectively.

**Table 2:** Descriptive statistics of reference gene expression in 5 *V*. *parahaemolyticus* strains for all growth temperatures by BestKeeper.

	*16S rRNA*	*gapdh*	*pvsA*	*pvuA*	*recA*	*rpoS*
Geometric mean [CP^1^]	8.85	19.13	27.79	26.93	22.23	19.70
Arithmetic mean [CP]	8.87	19.22	27.83	26.95	22.25	19.84
Minimum [CP]	8.15	15.58	24.95	24.42	20.55	16.27
Maximum [CP]	9.93	22.37	30.89	28.70	24.64	23.93
Standard deviation [+/- CP]^2^	0.45	1.49	1.20	0.97	0.87	2.00
Coefficient variation [% CP]	5.07	7.74	4.31	3.60	3.90	10.10
Minimum [x-fold]	-1.63	-11.71	-7.18	-5.68	-3.19	-10.78
Maximum [x-fold]	2.11	9.44	8.57	3.41	5.33	18.80
Standard deviation [+/- x-fold]	1.37	2.80	2.29	1.96	1.82	4.01

^1^CP = crossing point or threshold cycle (Ct).

^2^Genes can be ranked from the most stably expressed (lowest SD: *16S rRNA*) to the least stably expressed (highest SD: *rpoS*). Genes with SD [CP]>1 are considered to be inconsistent.

**Fig 2:**
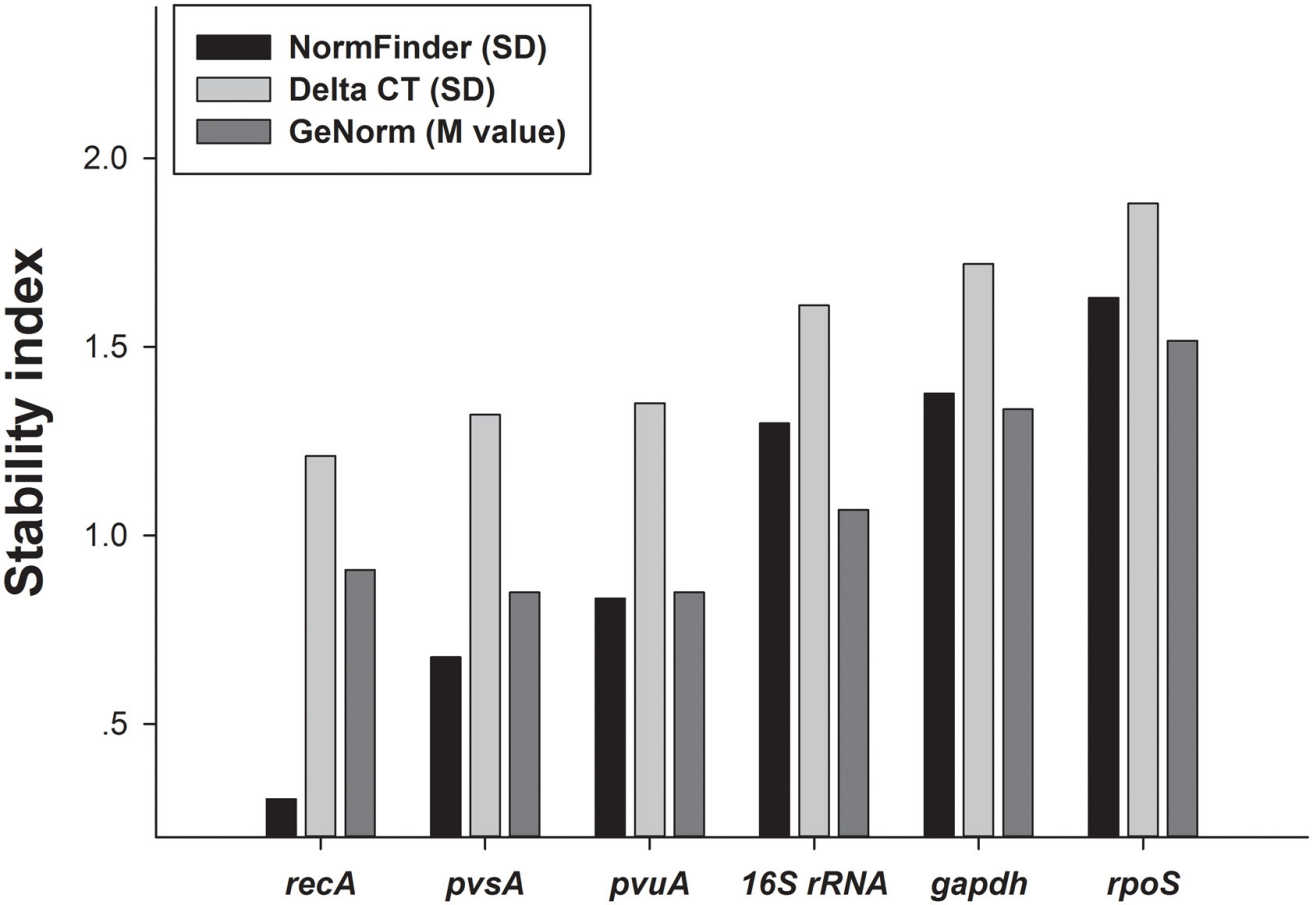
Analysis of reference gene stability by NormFinder, Delta CT, and GeNorm. For NormFinder and Delta CT, lower standard deviations (SD) values indicate more stable gene expression. For GeNorm, lower M values indicate more stable gene expression.

The NormFinder program was also used to rank the candidate reference genes according to expression stability across the strains and growth temperatures. The results (Fig 2) showed that *recA* and *pvsA* had the smallest variability, with SD of 0.301and 0.677, respectively, indicating that they were the most stable genes. The *pvuA* gene also showed low variability, with a SD of 0.833. The *gapdh* and *rpoS* genes were found to be the least stable genes, having the highest variability with stability values of 1.376 and 1.630, respectively. The Delta CT method resulted in a same ranking as that of NormFinder (Fig 2).

GeNorm program was also used to rank the expression of candidate genes according to the stability value, M value (Fig 2). The most stable reference genes across all growth temperatures were *pvsA* and *pvuA*, both with an M value of 0.849. The next most stable gene was *recA*, with an M value of 0.909. The *gapdh* and *rpoS* genes were the least stable genes with M values of 1.334 and 1.516, respectively.

**Fig 3:**
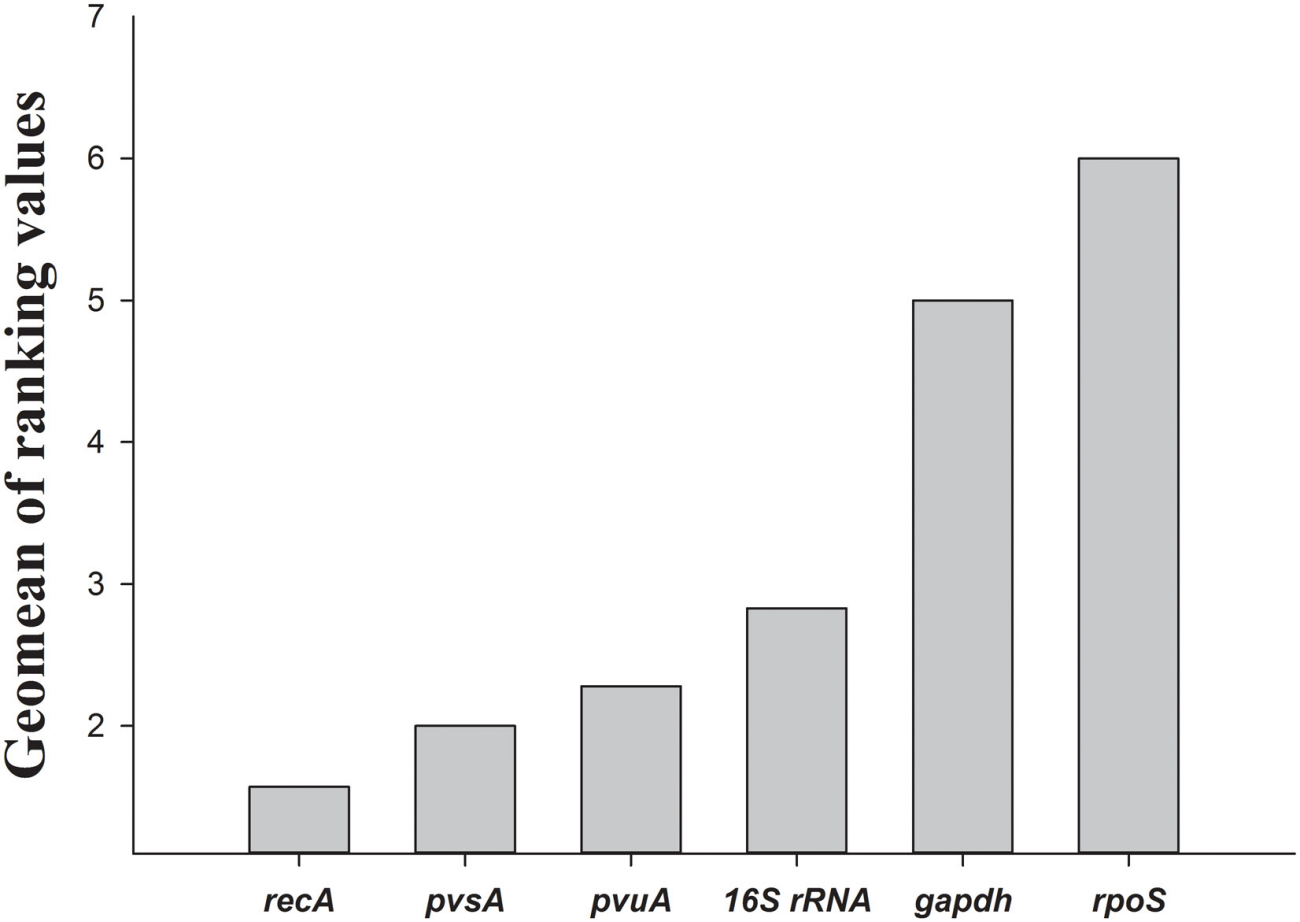
Analysis of reference gene stability by RefFinder. Ranking of candidate reference genes according to stability values produced by GeNorm, NormFinder, BestKeeper, and Delta CT, and the recommended comprehensive ranking calculated by RefFinder.

The expression stability of each gene in the each strain across the temperature was evaluated by RefFinder. The most stable gene was *recA* in *V*. *parahaemolyticus* ATCC33846, ATCC33847, and O3:K6, *pvuA* in ATCC17802, or *16S rRNA* in F13 (Table 3). At different growth temperatures, the most stable gene was *pvuA* in cells of *V*. *parahaemolyticu*s grown at 15°C, *pvsA* in cells grown at 37°C, and *16S rRNA* in cells grown at 42°C (Table 4). Finally, RefFinder calculated an overall final ranking and showed that *recA* was the most stably expressed gene for all strains and growth temperatures (Fig 3).

**Table 3:** Ranking of candidate reference genes according to the stability value of each *V*. *parahaemolyticus* strain for all growth temperatures. The stability measurements were produced by GeNorm, NormFinder, BestKeeper, and Delta CT, and the recommended comprehensive ranking was calculated by RefFinder.

Strain	Ranking Order (Better—Good—Average)					
	1	2	3	4	5	6
17802	*pvuA*	*pvsA*	*recA*	*16S rRNA*	*rpoS*	*gapdh*
33846	*recA*	*pvuA*	*pvsA*	*16S rRNA*	*rpoS*	*gapdh*
33847	*recA*	*pvuA*	*pvsA*	*16S rRNA*	*Gapdh*	*rpoS*
F13	*16S rRNA*	*pvsA*	*recA*	*gapdh*	*pvuA*	*rpoS*
O3:K6	*recA*	*pvuA*	*pvsA*	*16S rRNA*	*gapdh*	*rpoS*

**Table 4:** Ranking of candidate reference genes according the stability values of 5 *V*. *parahaemolyticus* strains at each culture temperatures for. The stability measurements were produced by GeNorm, NormFinder, BestKeeper, and Delta CT, and the recommended comprehensive ranking was calculated by RefFinder.

Temperature	Ranking Order (Better—Good—Average)					
	1	2	3	4	5	6
15°C	*pvuA*	*recA*	*pvsA*	*16S rRNA*	*gapdh*	*rpoS*
25°C	*recA*	*pvuA*	*16S* rRNA	*rpoS*	*pvsA*	*gapdh*
37°C	*pvsA*	*recA*	*rpoS*	*16S rRNA*	*pvuA*	*gapdh*
42°C	*16S rRNA*	*recA*	*gapdh*	*pvsA*	*pvuA*	*rpoS*

## Discussion

Housekeeping genes (HKGs) has been used as controls to normalize qRT-PCR. As a prerequisite, HKGs are very conserved in different conditions even in different bacterial species, and the expression of HKGs is often considered with lowest variability. However, literatures have reported that the expression of HKGs can vary with experimental conditions [13]. Therefore, reference genes need to be properly validated for their stability of expression for specific species, biological samples, and culture conditions, because accurate normalization of gene-expression levels is an absolute prerequisite for reliable results [23].

A direct analysis of the distribution of Ct values from qRT-PCR analyses cannot be used to rank candidate reference genes according to the stability of expression as it does not account for the efficiencies of PCR analysis. Several statistical algorithms have been developed to identify the stability of expression of reference genes. In this study, we used four common software programs (BestKeeper, NormFinder, GeNorm, and comparative Delta CT method) to determine the most stable reference genes in 5 *V*. *parahaemolyticus* strains grown at4 different temperatures. The BestKeeper algorithm uses Ct values directly to select the most stably expressed reference gene based on variations in the geometric means of Ct values [21]. The NormFinder and GeNorm algorithms use relative quantities derived from Ct values when calculating stability. NormFinder uses a model-based approach to evaluate the stability of individual reference genes while taking into account of variation across subgroups and avoids the artificial selection of co-regulated genes [20]. The GeNorm algorithm selects an optimal number of reference genes out of a larger group by selecting those showing the most similar expression across groups[15]. Comparative Delta CT method is a method similar to GeNorm, whereby “pairs of genes” are compared. This simple method bypasses the need to accurately quantify input RNA and instead uses Delta CT comparisons between genes [22]. At present, there is no consensus as to which of these algorithms should be used to select the ideal reference gene. However, based on the rankings from each program, RefFinder assigns an appropriate weight to individual genes and calculates the geometric means of their weights for the overall final ranking (http://www.leonxie.com/referencegene.php). The Delta CT and NormFinder programs showed a high level of consistency in ranking the most and least stable reference genes. Both Delta CT and NormFinder ranked *recA* and *pvsA* as the two most stable reference genes, while the GeNorm gave slightly different results, ranking *pvsA* and *pvuA* as the two most stable genes, while BestKeeper ranked *16S rRNA* as the most stable one. All four programs ranked *gapdh* and *rpoS* as the two least stable reference genes. These slight differences in ranking are to be expected as each program uses a distinct statistical algorithm to rank genes according to their stability.

Traditionally, ‘housekeeping’ genes such as *16S rRNA*, actin, glyceraldehyde 3-phosphate dehydrogenase (*gapdh*), and ubiquitin were used for data normalization. These genes were assumed to have a uniform level of expression due to their involvement in fundamental cellular processes. However, an evaluation of the expression stability of classical housekeeping genes has revealed unstable expressions in cells grown under different environmental conditions and growth stages and even different strains [24, 25]. Several reference genes have been shown to be more stably expressed than classical housekeeping genes. Hence, there is a need for systematic validation of internal reference genes in each organism and experiment. The reference gene candidates analyzed in this study were selected from genes previously used in qRT-PCR assays of *V*. *parahaemolyticus* and included *16S rRNA* [26], *recA* [25, 27], *rpoS* [28], *pvsA* [29], *pvuA* [29], and *gapdh* [24]. The results of this study identified *recA*, *pvuA*, and *pvsA* as the most stably expressed reference genes in *V*. *parahaemolyticus*. Part of the findings supports that *recA* as a common reference gene in several bacteria. The gene has been identified as a stable reference gene in *V*. *parahaemolyticus* [29], *Pectobacterium atrosepticum* [25], *Clostridium thermocellum* [30], *Enterococcus faecalis* [31], and *Streptococcus agalactiae* [24]. *pvuA* has also been identified as an appropriate reference gene in *V*. *parahaemolyticus* ATCC33847 strain that grown separately in seawater, filtered seawater,shrimp, and tryptone soya broth at 37°C [32]. The results from the present study of using 5 strains of *V*. *parahaemolyticus* and 4 growth temperatures provided stronger evidence that *pvuA* is a suitable reference gene in *V*. *parahaemolyticus*.

In this study, the reference genes were identified by analysis of 5 *V*. *parahaemolyticus* strains, O3:K6, ATCC33846, ATCC33847, ATCC17802, and F13. *V*. *parahaemolyticus* serotype O3:K6 was the most common serotype that caused outbreaks of diarrheal disease [33]. The ATTCC 33846 strain (O3 serotype) was isolated from patients with gastroenteritis in Japan. The ATCC 33847 strain (serotype unknown) was isolated from gastroenteritis illness in Maryland, USA. The ATCC 17802 strain (O1 serotype) was isolated from Shirasu food poisoning in Japan (http://www.atcc.org/). The F13 strain was isolated from shrimps in our laboratory. A recent review listed the possible cellular sensors, physiological parameters, and genetic regulators that allow bacteria to survive and adapt at different growth temperatures [34]. These strains may exhibit different physiology under stress conditions. For most bacterial species, there is lacking of an understanding of that which genes or gene sets are expressed during temperature downshifts [35]. To better understand the whole cell physiology, such as the expression of one or more genes using qPCR analysis under suboptimal growth conditions, additional studies are needed [35]. In this study, among the different strains of *V*. *parahaemolyticus* and growth temperatures, *recA* was the most stable reference gene in ATCC33846, ATCC33847, and O3K6 strains grown at 25°C, *pvuA* in ATCC17802 strain at 15°C, *16S rRNA* in F13 strain at 42°C, and *pvsA* in all strains at 37°C.

The expression of reference gene in prokaryotes has been shown to vary significantly with experimental conditions and the physiological state of the cell. The *V*. *parahaemolyticus* strains and growth temperatures used in this study were selected to allow for identification of reference genes that were stably expressed at different temperatures. The reference genes identified in this study are suitable for normalization of gene expression from different strains of *V*. *parahaemolyticus* as well as for *V*. *parahaemolyticus* strains grown at different temperatures. Therefore, the reference genes represent a robust set of genes that could be used for data normalization in a wide range of gene-expression studies in *V*. *parahaemolyticus*.

These results provide further evidence that expression of standard reference genes can be highly variable in prokaryotes depending on species and experimental conditions. Therefore, there is not a standard set of reference gene exists for gene expression studies in prokaryotes. This also highlights the importance of carrying out a reference gene stability study to select the most stable reference genes for a particular species under a given set of experimental conditions. This study examined 6 candidate genes, and *recA*, the most stably expressed reference gene identified in this study, is sufficient for the normalization of qRT-PCR data from *V*. *parahaemolyticus* of different strains and culture temperatures. The reference genes identified in this study may provide a starting point for selection of candidate reference genes for gene expression studies in other related species.

## Supporting Information

S1 File Specificity of qRT-PCR amplification. Melting curves for candidate reference genes (Figure A). Agarose gel (1%) showing amplification of a single product of expected size for each candidate reference gene (Figure B).
(TIF)

S1 Table Data for the Figures. (XLSX)
