# Supplementary figures and images for: Investigation of Reference Genes in Vibrio parahaemolyticus for Gene Expression Analysis Using Quantitative RT-PCR
Source: PLoS One. 2015 Dec 11;10(12):e0144362. doi: 10.1371/journal.pone.0144362 (PMC4676679; doi:10.1371/journal.pone.0144362)

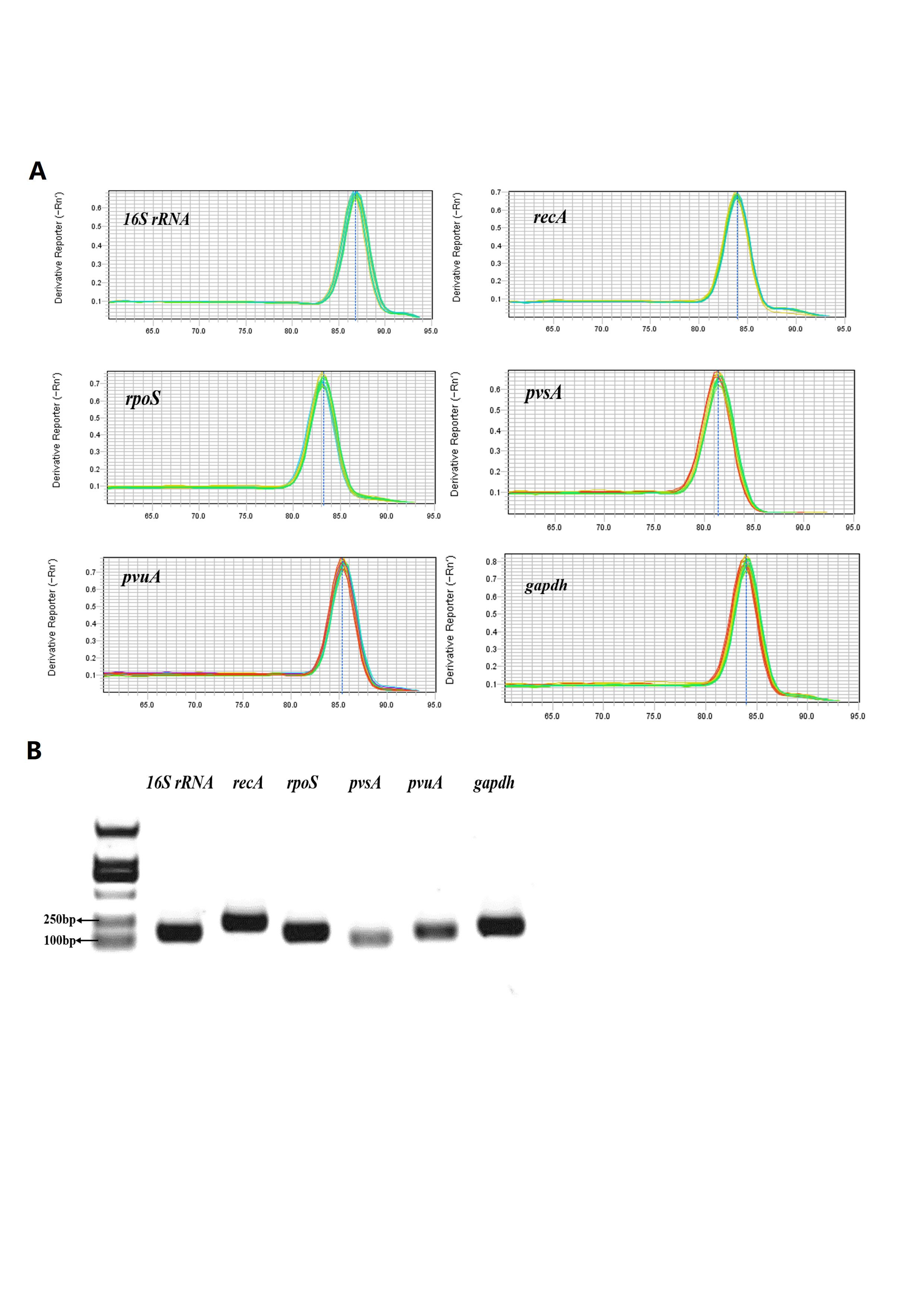

Supplement: S1 File — Melting curves for candidate reference genes (Figure A). Agarose gel (1%) showing amplification of a single product of expected size for each candidate reference gene (Figure B). (TIF) [file pone.0144362.s001.tif]
